# Rapid diagnosis of lymph node metastasis in breast cancer using a new fluorescent method with γ-glutamyl hydroxymethyl rhodamine green

**DOI:** 10.1038/srep27525

**Published:** 2016-06-09

**Authors:** Yoshiaki Shinden, Hiroki Ueo, Taro Tobo, Ayako Gamachi, Mitsuaki Utou, Hisateru Komatsu, Sho Nambara, Tomoko Saito, Masami Ueda, Hidenari Hirata, Shotaro Sakimura, Yuki Takano, Ryutaro Uchi, Junji Kurashige, Sayuri Akiyoshi, Tomohiro Iguchi, Hidetoshi Eguchi, Keishi Sugimachi, Yoko Kubota, Yuichiro Kai, Kenji Shibuta, Yuko Kijima, Heiji Yoshinaka, Shoji Natsugoe, Masaki Mori, Yoshihiko Maehara, Masayo Sakabe, Mako Kamiya, John W. Kakareka, Thomas J. Pohida, Peter L. Choyke, Hisataka Kobayashi, Hiroaki Ueo, Yasuteru Urano, Koshi Mimori

**Affiliations:** 1 Department of Surgery, Breast and Thyroid Surgery, Kagoshima University, Graduate School of Medical and Dental Sciences, 8-35-1, Kagoshima-City, Kagoshima 890-8520, Japan; 2 Department of Digestive Surgery, Breast and Thyroid Surgery, Kagoshima University Graduate School of Medical and Dental Sciences, Kagoshima 890-8520, Japan; 3 Department of Surgery and Sciences, Graduate School of Medical Sciences, Kyushu University, Fukuoka 812-8582, Japan; 4 Department of Pathology, Kyushu University Beppu Hospital, 4546 Tsurumihara, Beppu 874-0838, Japan; 5 Department of Pathology, Oita University, Yufu 879-5593, Japan; 6 Ueo Breast Surgery Hospital, Oita 870-0854, Japan; 7 Department of Gastroenterological Surgery, Graduate School of Medicine, Osaka University, Suita 565-0871, Japan; 8 Graduate School of Medicine, The University of Tokyo, Tokyo 113-0033, Japan; 9 Signal Processing and Instrumentation Section, Division of Computational Bioscience, Center of Information Technology, National Institutes of Health, Bethesda, MD 20892-5624, USA; 10 Molecular Imaging Program, Center for Cancer Research, National Cancer Institute, National Institutes of Health, Bethesda, MD 20892-1088, USA; 11 CREST, AMED, Tokyo 100-0004, Japan

## Abstract

Sentinel lymph node biopsy is performed as a standard procedure in breast cancer surgery, and the development of quick and simple methods to detect metastatic lesions is in high demand. Here, we validated a new fluorescent method using γ-glutamyl hydroxymethyl rhodamine green to diagnose metastatic lymph nodes in breast cancer. One hundred and forty-nine lymph nodes from 38 breast cancer patients were evaluated in this study. Comparison of fluorescent and pathological images showed that this fluorescent method was successful for visualizing breast cancer cells in lymph nodes. This method had a sufficiently high sensitivity (97%), specificity (79%) and negative predictive value (99%) to render it useful for an intraoperative diagnosis of cancer. These preliminary findings suggest that this novel method is useful for distinguishing non-cancerous specimens from those in need of careful examination and could help save time and cost for surgeons and pathologists.

Sentinel lymph node (SLN) biopsy (SLNB) has been performed as a standard procedure in breast cancer surgery^1^,^2^,^3^,^4^. SLNB can be used to predict metastasis to the axillary lymph nodes with high accuracy, and it precludes the removal of axillary lymph nodes and the subsequent complications associated with axillary clearance in node-negative breast cancer patients^4^,^5^.

SLN metastasis is usually diagnosed by intraoperative pathological observation of hematoxylin and eosin (H&E)-stained frozen sections and cytological observation of touch imprints followed by definitive postoperative histopathological examination of permanent sections^1^,^6^. Issues associated with these intraoperative methods include insufficient sensitivity^5^ and long processing times^6^. Recently, one-step nucleic acid amplification (OSNA) has arisen as a new molecular technique^5^. OSNA has high sensitivity and specificity in SLNB; however, it is a relatively costly pathological technique^7^, and some reports demonstrated its low positive predictive value^8^,^9^. Regardless of the technological achievements, we still require rapid and convenient methods to detect cancer cells in the lymph nodes and to diagnose metastasis to the SLN intraoperatively.

We previously developed γ-glutamyl hydroxymethyl rhodamine green (gGlu-HMRG) as a tool to detect viable cancer cells, based on the fact that the γ-glutamyltranspeptidase (GGT) enzyme is overexpressed in the membranes of various cancer cells but not in normal tissue^10^. Recently, we disclosed the usefulness of gGlu-HMRG in the identification of tumor tissues surrounded by normal breast tissue and by fat. We showed that this new gGlu-HMRG-based fluorescent technique is applicable for intraoperative margin assessment during breast conserving surgery^11^.

In this study, we extended the application of a gGlu-HMRG-based fluorescent method to the evaluation of resected lymph nodes and determined its clinical significance as a diagnostic tool for metastatic lymph nodes.

## Materials and Methods

### Clinical samples

Breast cancer patients (n = 38) who underwent surgical treatment at two hospitals (Kyushu University Beppu Hospital and Ueo Breast Surgery Clinic) from 2012 to 2013 were enrolled in this study. Before sample acquisition, each patient provided written informed consent at the respective hospital. The ethics committees of Kyushu University approved this study, and all experimental methods were carried out in accordance with the approved guidelines. No patients received neo-adjuvant chemotherapy. Preoperative clinical information, including lymph node metastases, was obtained by mammography, ultrasound and computed tomography and/or magnetic resonance imaging. SLNB was conducted in patients diagnosed as negative for lymph node metastases preoperatively using patent blue dye. Axillary lymph node dissection was conducted in patients diagnosed as positive for lymph node metastases preoperatively or diagnosed as positive for lymph node metastases intraoperatively after SLNB. The clinicopathological information is listed in Table 1. One hundred and forty-nine lymph nodes from 38 patients were examined. Ninety-one lymph nodes (32 metastatic lymph nodes) were resected from 18 patients who were diagnosed with metastatic lymph nodes preoperatively and underwent removal of axillary lymph nodes. Fifty-eight lymph nodes were resected from 20 patients who were not diagnosed with metastatic lymph nodes preoperatively and underwent SLNB (Supplementary Figure 1).

### Detection of fluorescence

Each lymph node was sliced at its maximum diameter immediately after resection, and 1 ml gGlu-HMRG (50 μM, containing 0.5% v/v DMSO as a co-solvent) was added to the surface of each specimen. Fluorescence was measured using an in-house fluorescence camera unit^12^. Four blue light-emitting diodes (LEDs) and narrow band pass optical filters were mounted in front of each LED assembly emitting excitation light at 480 nm with a bandwidth of 30 nm. The fluorescent light was detected by an off-the-shelf color charge-coupled device camera and a long-pass emission filter in front of the lens. Snapshot images were used to calculate the fluorescent intensities (FI). Each image was recorded as pixel intensity values in the range of 0 to 255.

### Analysis of fluorescent images

To evaluate the fluorescence in the resected lymph nodes, a 200-μm-diameter circular region was set as the region of interest (ROI) in each lymph node fluorescent image to ensure detection of micrometastasis. We calculated the average FI in each ROI and determined the increase in FI by subtracting the mean intensity measured just after gGlu-HMRG administration from the mean intensity 5 minutes post-application. All fluorescent images were analyzed using Image J software (National Institutes of Health, Rockville, MD, USA) (http://rsbweb.nih.gov/ij/).

### Statistical analysis

Statistical analyses were performed using JMP Pro 9.0.2 for Mac OS (SAS Institute Inc., Cary, NC, USA). The Mann-Whitney test was used to compare mean FI values. A P value < 0.05 was considered to indicate a statistically significant difference. Differences between clinicopathological factors were analyzed by χ^2^ test for categorical variables and by t-test for continuous variables.

## Results

### The detection of cancer cells in lymph nodes using the gGlu-HMRG-based fluorescent method

**Figure 1:**
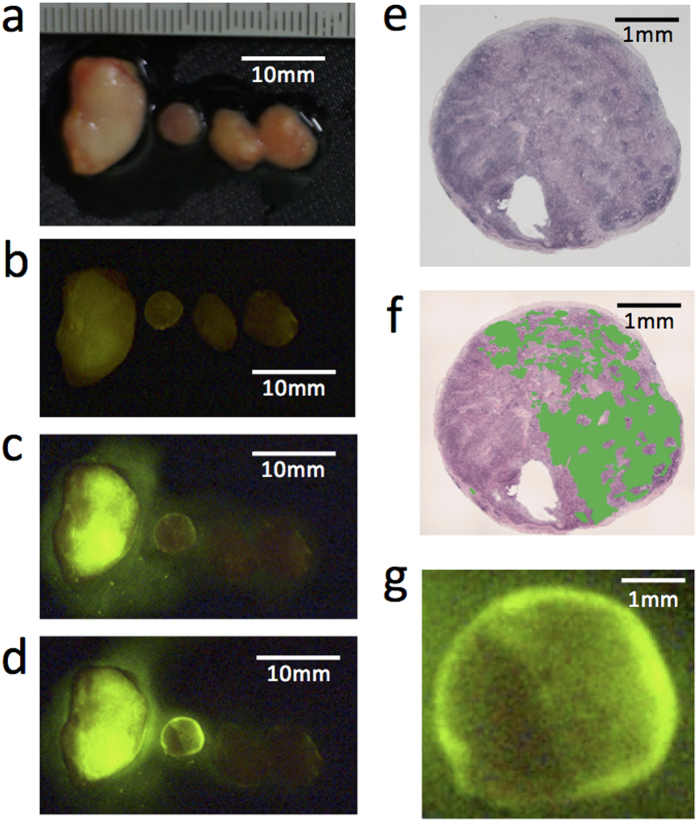
Detection of cancer cells in lymph nodes using gGlu-HMRG fluorescence. (**a**) Macroscopic image of resected lymph nodes from one breast cancer surgery case. (**b**) Fluorescent image of the same lymph nodes as in (**a**) before administration of gGlu-HMRG. Autofluorescence is indicated by the faint green color. Fluorescent images (**c**) 5 minutes and (**d**) 15 minutes after administration of gGlu-HMRG. (**e,f**) H&E staining of the same lymph node second from the left in (**a–d**)). This lymph node was diagnosed pathologically as metastatic. (**f**) Metastatic regions were indicated by green color. (**g**) Magnified fluorescent image of the same lymph node in (**e,f**) 15 minutes after administration of gGlu-HMRG.

A time-dependent increase in FI was observed in metastatic lymph nodes (Fig. 1). Although metastatic lymph nodes are usually enlarged and solid, some are macroscopically small and soft. This fluorescent method was capable of distinguishing small cancer regions in the macroscopically normal lymph nodes (the second lymph node from the right in Fig. 1a–d). According to the fluorescent and H&E-stained images, there was good accordance between the fluorescent regions and metastatic lesions in the lymph nodes (Fig. 1e–g). The results suggested that this gGlu-HMRG-based fluorescent method enables the identification of candidate metastatic lymph nodes for dissection.

### Diagnosis of metastatic lymph nodes using the gGlu-HMRG-based fluorescent method

**Figure 2:**
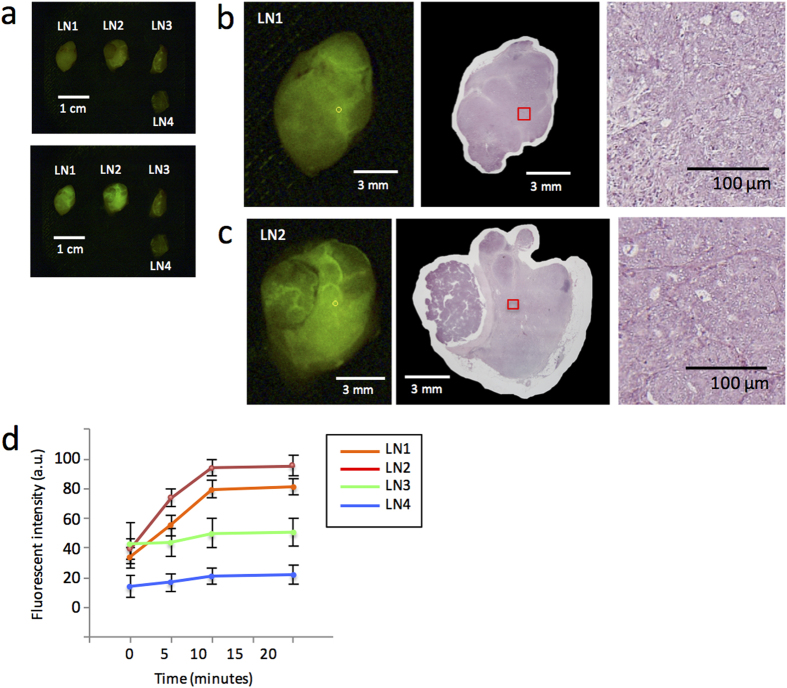
Detection of metastasis in lymph nodes using gGlu-HMRG fluorescence. (**a**) Fluorescent image of four resected lymph nodes before (upper picture) and 15 minutes after (bottom picture) administration of gGlu-HMRG. (**b,c**) Fluorescent images 15 minutes after administration of gGlu-HMRG (left) and H&E staining (middle) of two metastatic lymph nodes. The small yellow circles on the left indicate the ROIs that showed the strongest increase in fluorescent intensity within 5 minutes. The small red boxes in the middle images correspond with the yellow circles on the left. Magnified H&E staining of the regions within the red boxes are shown on the right, and these were diagnosed as cancerous lesions by pathological examination. (**d**) Time-dependent increases in the fluorescent intensity of each lymph node after administration of gGlu-HMRG.

**Figure 3:**
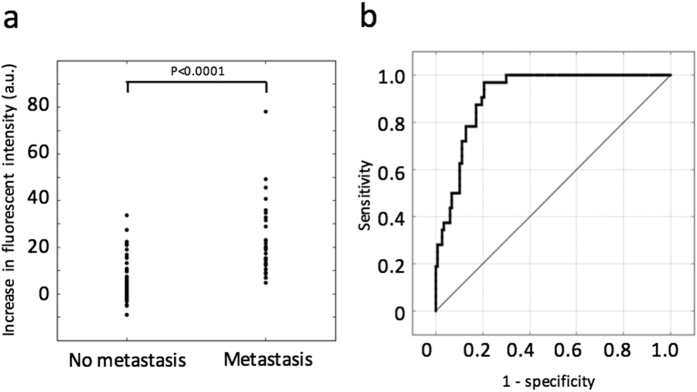
Diagnosis of metastatic lymph nodes using gGlu-HMRG fluorescence. (**a**) The increase in fluorescent intensity in metastatic versus non-metastatic lymph nodes. Significant differences in fluorescence were observed. (**b**) A receiver operating characteristics curve for pathological metastatic diagnostic discrimination using the fluorescent method.

Next, we assessed the diagnostic accuracy of the gGlu-HMRG-based fluorescent method for intraoperative diagnosis of lymph node metastasis in breast cancer. One example of the evaluation of fluorescent images, including two metastatic and two non-metastatic lymph nodes, is shown in Fig. 2a–c. For quantification of the increase in FI, the ROI showing the strongest increase in intensity was selected from each lymph node, and FI was measured in a time-dependent manner within that ROI (Fig. 2d). The two metastatic lymph nodes showed more of an increase in FI than that of the two non-metastatic lymph nodes. Especially, a significant increase in FI was observed within 5 minutes. The increase in FI detected after 5 minutes was compared between the metastatic and non-metastatic lymph nodes and was significantly greater in the former (Fig. 3a). When we set a threshold value of 6.8 arbitrary unit (a.u.), the sensitivity and specificity were 97% and 79%, respectively. The positive and negative predictive values were 56% and 99%, respectively (Table 2). The receiver operating characteristics curve is shown in Fig. 3b.

The negative predictive value of this method was so high that pathological examination for fluorescence-negative sentinel lymph nodes in SLNB was not necessary. In this study, 58 lymph nodes from 20 patients were resected by SLNB and diagnosed as non-metastatic lymph nodes intraoperatively. Of those 58 non-metastatic lymph nodes, 13 from 8 patients were positive for fluorescence. In the remaining 12 patients, all lymph nodes were negative for fluorescence and pathologically non-metastatic.

### The relationship between clinicopathological information and fluorescence

In this study, we detected 24 non-metastatic lymph nodes with false-positive fluorescence among 11 patients. To examine the reason for the false-positive fluorescence, we compared clinicopathological factors between patients with at least one fluorescence-positive lymph node versus no fluorescence-positive lymph nodes (Table 3). We could not identify factors correlating with fluorescence, other than histology (invasive ductal carcinoma or DCIS) and the T factor. This was partially due to the tendency of more metastatic lymph nodes to be present in large tumors than small tumors and in invasive ductal carcinoma than DCIS (Table 1). We could not clarify the reason for false-positive fluorescence; however, it is supposed that this fluorescent method can be applied universally to breast cancer and its various subtypes, because gGlu-HMRG fluorescence is not correlated with the expression of the estrogen, progesterone or human epidermal growth factor 2 receptors.

## Discussion

In this study, we assessed the intraoperative diagnostic ability of a gGlu-HMRG-based fluorescent method for metastatic lymph nodes during breast cancer surgery.

This fluorescent method is a quick and easily performed procedure, thereby saving time and cost for surgeons and pathologists. In breast cancer surgery, intraoperative SLNB is widely performed, and thus such methods for SLNB are in much demand. For pathological evaluation of SLN in breast cancer, it is recommended to slice SLN no thicker than 2.0 mm to prevent overlooking macrometastases^13^. As a result, extra time and cost may be required to mount, stain and examine large resected lymph node specimens pathologically. Because our method had a high sensitivity (97%), it may be useful for discriminating non-cancerous sections from those deserving careful examination. It may minimize the difficulties encountered with sampling and reduce the burden to pathologists. Additionally, because our method had a high negative predictive value (99%), it precludes a pathological examination in cases negative for fluorescence, thereby saving significant time and cost.

Recently, OSNA has arisen as a new intraoperative diagnosing technique. OSNA has advantages of high sensitivity (78.1–100%) and specificity (93.4–99.3%); however, OSNA requires time for processing lymph node specimens and 36–46 minutes to diagnose one to three nodes^14^. By contrast, approximately 5 to 10 minutes are required for measurement using our method. When the fluorescence is negative according to our method, this is sufficient for the intraoperative diagnosis, and a pathological examination is not necessary. In the analysis of 20 SNLB cases, we diagnosed 12 cases as negative lymph node metastasis. Therefore, more than half of SLNB cases are estimated to benefit from this method. If fluorescence is positive, additional pathological examination is needed; however, we could select those slices for microscopic examination to reduce the burden of pathologists. In addition, our fluorescent method is non-destructive and can harmonize with conventional pathological diagnosis. This method can be used to detect various subtypes and histopathological types of breast cancer and is expected to be applied widely.

Previously, we reported that this novel fluorescent method could detect various cancer cells in mice and human breast cancer cells *ex vivo*^10^,^11^. This method had high sensitivity and specificity in primary breast tumors; therefore, we expected a high rate of accuracy in detecting lymph node metastasis as well via this method. Despite high sensitivity, this fluorescent method had relatively low specificity (79%) in the diagnosis of metastatic lymph nodes compared with primary tumors in our previous study. In some specimens, a moderate increase in FI was detected in non-metastatic lymph nodes. In a pathological examination using H&E staining, no specific characteristics were observed in non-malignant fluorescence-positive lymph nodes. It was assumed that the GGT protein from primary tissue is secreted into lymph fluid where it reacts with gGlu-HMRG; however, none of the pathological factors significantly correlated with false fluorescent positivity were detected in patients without lymph nodes metastasis. Further experiments regarding false-positive fluorescence should be examined in the future.

A limitation of this study is that we did not evaluate any metastatic lymph nodes with micrometastases or any isolated tumor cells. For micrometastatic foci (≦2 mm) in SLN, recent reports recommended avoiding axillary dissection^15^,^16^,^17^,^18^,^19^. Therefore, it is desirable to distinguish macrometastases from micrometastases and isolated tumor cells (≦0.2 mm) for intraoperative lymph node diagnosis during breast cancer surgery. In this study, we placed importance on the detection of cancer cells in lymph nodes and set ROIs of 0.2 mm in diameter to prevent overlooking micrometastasis. In the future, this method of analysis is needed for discriminating macrometastases and micrometastases.

We present a new application for the intraoperative diagnosis of lymph node metastases using gGlu-HMRG. This technique is promising in breast cancer surgery.

## Additional Information

**How to cite this article**: Shinden, Y. *et al.* Rapid diagnosis of lymph node metastasis in breast cancer using a new fluorescent method with γ-glutamyl hydroxymethyl rhodamine green. *Sci. Rep.* **6**, 27525; doi: 10.1038/srep27525 (2016).

## Supplementary Material

Supplementary Information

## Figures and Tables

**Table 1:** Clinicopathological information of the enrolled patients.

Factors	All patients (n = 38)		Without lymph node metastasis (n = 27)		With lymph node metastasis (n = 11)		p value
	number	%	number	%	number	%	
Age (mean)	59.6		63 ± 11		58 ± 14		0.15
T							
is-1	21	55	17	63	4	36	0.13
2–4	17	45	10	37	7	64	
Histology							
Invasive ductal carcinoma	27	71	17	63	10	91	0.055
Papillo-tubular	6		6		0		
Solid-tubular	8		5		3		
Scirrhous	8		4		4		
Other	5		2		3		
DCIS	7	18	7	26	0	0	
Special types	4	11	3	11	1	9	
HER2 receptor							
Positive	6	20	2	11	4	36	0.09
Negative	24	80	17	89	7	64	
Lymph node metastasis							
Absent	27	71	7	41	1	10	0.09
Present	11	29	10	59	9	90	
Lymphatic invasion							
0, 1	27	90	18	95	9	82	0.26
2, 3	3	10	1	5	2	18	
Venous invasion							
0, 1	30	100	19	100	11	100	
2, 3	0	0	0	0	0	0	
Operation							
Mastectomy + ALND	17	45					
Mastectomy + SLNB	3	8					
BCS*** + ALND	1	3					
BCS + SLNB	17	45					

*T factor was assessed according to the TNM Classification of Malignant Tumors, 7^th^ edition^20^.

**Not available for one case, and no examination was performed for seven DCIS cases.

^***^Breast conserving surgery.

**Table 2:** Evaluation of the gGlu-HMRG-based fluorescent method for diagnosis of metastatic lymph nodes in breast cancer.

Pathological findings			
	Metastasis	Non-metastasis	
Fluorescence-positive	31	24	55
-negative	1	93	94
	32	117	

**Table 3:** Clinicopathological information of the patients with fluorescence-positive and -negative lymph nodes.

Factors	Fluorescence-positive (n = 22)		Fluorescence-negative (n = 16)		p value
	number	%	number	%	
Age (mean ± SD*1)	61 ± 11		58 ± 16		0.28
Histology					
Invasive ductal carcinoma	19	86	8	50	0.043*
Papillo-tubular	2		4		
Solid-tubular	7		2		
Scirrhous	6		2		
Other	4		0		
DCIS	2	9	5	31	
Special types	1	5	3	19	
T					
Is-1	8	36	11	69	0.049
2–4	14	64	5	31	
Estrogen receptor					
Positive	19	86	12	75	0.37
Negative	3	14	4	25	
Progesterone receptor					
Positive	17	77	12	75	0.87
Negative	5	23	4	25	
HER2 receptor					
Positive	5	25	1	10	0.33
Negative	15	75	9	90	
Lymphatic invasion					
0, 1	17	85	9	100	0.70
2, 3	3	15	1	0	
Venous invasion					
0, 1	20	100	10	100	
2, 3	0	0	0	0	

^*^Invasive ductal carcinoma vs. DCIS.
