# Supplementary material for: Rapid diagnosis of lymph node metastasis in breast cancer using a new fluorescent method with γ-glutamyl hydroxymethyl rhodamine green
Source: Sci Rep. 2016 Jun 9;6:27525. doi: 10.1038/srep27525 (PMC4899706; doi:10.1038/srep27525)
Supplement: Supplementary Information [file srep27525-s1.pdf]

Title: Rapid diagnosis of lymph node metastasis in breast cancer using a new fluorescent method with γ-glutamyl hydroxymethyl rhodamine green

Authors: Yoshiaki Shinden[1, 5]*, Hiroki Ueo[1, 7]*, Taro Tobo[2], Ayako Gamachi[3], Mitsuaki Utou[2], Hisateru Komatsu[1], Sho Nambara[1], Tomoko Saito[1], Masami Ueda[1], Hidenari Hirata[1], Shotaro Sakimura[1], Yuki Takano[1], Ryutaro Uchi[1], Junji Kurashige[1], Sayuri Akiyoshi[1], Tomohiro Iguchi[1], Hidetoshi Eguchi[1], Keishi Sugimachi[1], Yoko Kubota[4], Yuichiro Kai[4], Kenji Shibuta[4], Yuko Kijima[5], Heiji Yoshinaka[5], Shoji Natsugoe[5], Masaki Mori[6], Yoshihiko Maehara[7], Masayo Sakabe[8], Mako Kamiya[8], John W. Kakareka[9], Thomas J. Pohida[9], Peter L. Choyke[10], Hisataka Kobayashi[10], Hiroaki Ueo[4], Yasuteru Urano[8, 11] and Koshi Mimori[1]

* These authors contributed equally to this work.

Figure legend

Supplementary figure 1: A flow chart depicting the selection of the enrolled patients and evaluation of the lymph nodes.

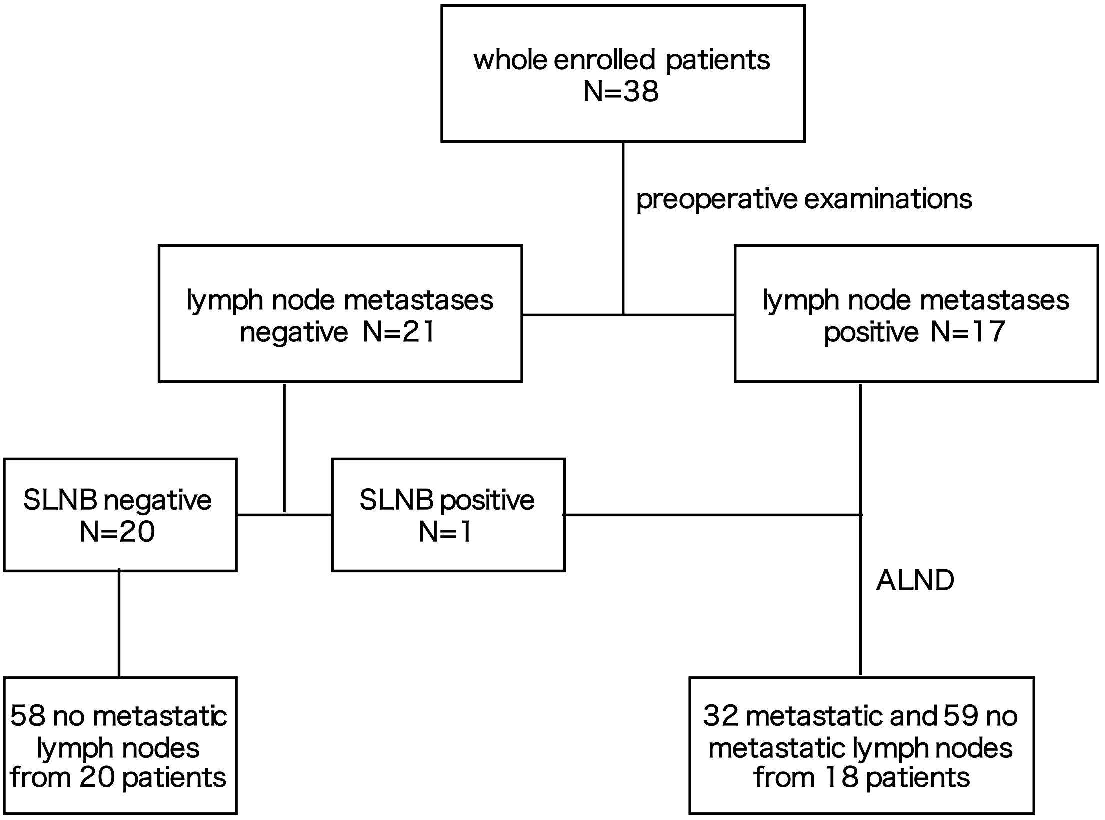
whole enrolled patients
N=38
preoperative examinations
lymph node metastases negative N=21
lymph node metastases positive N=17
SLNB negative N=20
SLNB positive N=1
ALND
58 no metastatic lymph nodes from 20 patients
32 metastatic and 59 no metastatic lymph nodes from 18 patients
